# The Experimental Composition Improvisation Continua Model: A Tool for Musical Analysis

**DOI:** 10.3389/fpsyg.2021.611536

**Published:** 2021-03-23

**Authors:** Alister Spence

**Affiliations:** Lecturer in Music, School of Arts and Media, The Faculty of Arts, Design and Architecture, University of New South Wales, Sydney, NSW, Australia

**Keywords:** music improvisation, music composition, composition-improvisation continuum, contingency, experimental music, musical creativity, music performance

## Abstract

Among improvisers and composers today there is a resurgence of interest in experimental music (EM) practices that welcome contingency; engaging with unforeseen circumstances as an essential component of the music-making process, and a means to sonic discovery. I propose the *Experimental Composition Improvisation Continua* (ECIC) as a model with which to better understand these experimental musical works. The historical Experimental Music movement of the 1950s and 60s is briefly revisited, and the jazz tradition included as an essential protagonist; both being important historical movements leading to the formulation of ideas around contingent musical practices. The ECIC model is outlined as providing a means to observe the interactions and continua between composition and improvisation on the one hand and more or less experimentally conceived music on the other. This model is applied as an investigative and comparative tool to three distinctive works in order to illuminate the presence or otherwise of various experimental interactions within them. The works are: “Spiral Staircase” – a composition by written by Satoko Fujii in late 2007, John Cage’s *4′33″*, and a performance of “My Favorite Things” by the John Coltrane Quartet. Further possible applications of the ECIC are suggested in the conclusion.

## Introduction

Among improvising musicians today, and composers who are writing for improvisers, there is a burgeoning interest in experimental music (EM) practices that transcend idiom and musical tradition (Beins et al., 2011; Cox and Warner, 2013; Gottschalk, 2016). I am referring to music making that actively engages with unforeseen circumstances and outcomes as an essential component of the work (Nyman, 1999, 1–30).^1^ Although often operating on the fringes of musical communities, the enormous output of this work, *via* concerts and recordings, has been reviewed widely by a host of music magazines and on-line blogs. At the same time, with a few notable exceptions,^2^ little has been documented or analyzed in contemporary scholarly writing that examines the field in a holistic manner or takes into account either its breadth of practice or its effective cross-genre artistic contribution. This article investigates the active commonalities and convergences in experimental compositional and improvisational work *across* stylistic delineations. Connections are highlighted between the action of chance and indeterminism in improvisation, and its associated relationships with composition and a model introduced as an investigative tool. The environment of contingency, chance, and indeterminism is proposed as a catalyst for new ideas, new interactions, new sounds, and new perceptions. What I have termed the *Experimental Composition Improvisation Continua* (ECIC) view the relationships between the experimental, compositional, and improvisational elements in music making as being in flux: flowing between differing degrees of engagement (Spence, 2018, 2020). The ECIC model offered in this article is a qualitative tool with which to investigate and compare experimental, compositional, and improvisational work – both historical and contemporary – beyond genre, and to identify the contribution of elements within this work to unforeseen outcomes.^3^ The model can be used to compare the musical actions and outcomes of composers, performers, and the musical environment, both within a work and across works.

I take the Experimental Music Movement in the 1950s and 60s as a starting point for the crystallization of “beyond idiom” ideas around contingency and chance. I also acknowledge the now well-documented debt that EM owes to jazz music as highlighted in the writings of Lewis (1996, 2009), Radano (2009), Kim (2012), and others.

## Aims

In this article, I identify the nature and activity of experimentalism, composition, and improvisation in music, and the relationships and continua between them and across musical style. I propose the ECIC as an investigative and comparative tool with which to study these elements in experimental music and apply this tool to “Spiral Staircase,” a piece performed by the Satoko Fujii Quartet, in order to demonstrate its application. Additionally, I suggest further applications of the ECIC tool and use two well-known examples: the composition *4′33″* by John Cage, and a recorded performance of “My Favorite Things” by John Coltrane in the way of demonstration (Coltrane, 2007).

The questions this article investigates are:

- What characterizes “Experimental Music,” “Composition,” and “Improvisation,” and what are the relationships and continua between them?
- How can these elements be addressed in combination in an investigative tool for experimental music?
- How can the ECIC model be practically utilized to investigate and compare interactions and positionings between experimentalism, composition, and improvisation in live and recorded performance contexts, and also as a means to reflect on pre-existing – for instance compositional – musical processes?

## Literature Review: Background and ECIC Relationships

### Experimental Music

The Experimental Music (EM) movement was a development within American Art music that was formalized in New York between 1950 and 1951, through the work of composers John Cage, Earle Brown, Morton Feldman, Christian Wolff, and others. These composers explained the focus of EM as being concerned with sound for its own sake, free from historical and traditional associations. They investigated new relationships between sonorities by utilizing processes that caused or allowed accidents, randomness, illusions, or problems, to be negotiated by the composer and/or the performer and/or the audience. Morton Feldman says of this, “only by ‘unfixing’ the elements traditionally used to construct a piece of music could the sounds exist in themselves – not as symbols, or memories which were memories of other music to begin with” (Feldman and Friedman, 2000, 35). When publicly introducing the term in 1955, Cage said of EM “the word ‘experimental’ is apt, providing it is understood not as descriptive of an act to be later judged in terms of success and failure, but simply as of an act the outcome of which is unknown” (Cage, 2010, 13). In the African American improvising community, the search for new sound through chance, indeterminism, and contingent process was being explored concurrently, resulting in the “Free Jazz” movement which emerged in the mid-1950s (Jost, 1994; Lewis, 2009). Sun Ra, Ornette Coleman, Cecil Taylor, John Coltrane, Archie Shepp, Muhal Richard Abrams, and others pioneered a new and exploratory form of improvising that engaged with chance elements through practices and processes such as group improvisation, open unstructured forms, and freedom of choice with regard to tonality. The democratic, non-hierarchical aspects of group interaction, the relationship to motoric rhythm, and the focus on personal narrative marked this music as a distinctive expression of experimentalism. Musician and academic George Lewis terms this expression as *Afrological* experimentalism (Lewis, 1996). Lewis also contends that the bebop development in jazz music, which unfolded in the 1940s, was at its core experimental in nature. He says of the relationship between EM and improvisation “indeterminacy could well be not a successor to improvisation but a subset of it” (1996, 229). In other words, Lewis is saying that the EM and jazz traditions have direct links *via* the processes and practices of improvisation. However, at the time, these links were not clearly established. Lewis (1996, 222) describes what he sees as a deliberately manufactured divide between Afrological and *Eurological* experimentalism saying that Eurological experimental “composers such as Cage and Feldman located their work as an integral part of the sociomusical art world that explicitly bonded with the intellectual and music traditions of Europe,” and that there was “an ongoing narrative of dismissal, on the part of many of these [Eurological] composers, of the tenets of African-American improvisative forms” (216).^4^ While John Cage was outspoken regarding his suspicion of the connection between jazz music and experimentalism, other experimental music composers, such as Earle Brown, Terry Riley, and La Monte Young recognized the connections and, to some extent, acknowledged them in their practice (These three composers all had experience as jazz performers prior to or early in their careers, with Riley and La Monte Young continuing to apply their improvisatory skills in their EM practice).^5^ The range of experimental practices and processes that were investigated in the 1950s and 60s – both within Jazz and the Experimental Music movement – in America and Europe included: indeterminism of pitch (and timbre) and time,^6^ graphic scores, instrumental preparations, Musique Concrète, and other “acousmatic” practices (Schaeffer, 1966; translated, 2017, 64–69), utilizing electronic sound recording and producing media, sound “theater” (see, for instance, the work of the art movement Fluxus, in Nyman, 1999, 72–88), solo and group improvisation (guided and unguided), and minimalism (acoustic and electronic).^7^ These practices continue by in large to be those that are being investigated today, albeit with updated technological tools. Books such as *Audio Culture* (Cox and Warner, 2013) document the adaptation and ongoing development of experimental musical practices and processes, and a range of books outline experimental work in specific locations, “scenes,” and cultures (Plourde, 2008; Saunders, 2009; Beins et al., 2011; Piekut, 2014; Toop, 2016). Enduring experimental musical expressions are emerging within and across classical/art music, jazz, rock, folk-musics, free improvisation, sound art, electro-acoustic music, noise music, and the plethora of genres and sub genres that categorize contemporary music. These works are proving protectionist debates regarding experimentalism – such as those regarding process, genre, style, and music culture – to be irrelevant.^8^ As a term “experimental music,” like many category terms in music (such as “classical music,” or “jazz”), is somewhat problematic. While it was first used to describe a particular development in American classical music, it continues to be used as a category description and, as such, has attracted some criticism and semantic deviation. Landy (1991, 6), for example, has argued that in his view “purposelessness” is stated by EM musicians as one of their intentions, and that “any good definition of *experiment* shows that purposelessness is by no means an experimental goal.” Joanne Demers aligns with a more recent popularist view of equating experimental music with the avant-garde, as a “series of unusual practices whose strangeness stands out in relation to whatever the mainstream happens to be” (Demers, 2010, 7). This is a view detached from connections with the historical EM movement and its essential link with contingency, chance, or the “unknown.” It is my contention that the historically situated term “experimental music” remains a useful and relevant descriptor for an approach to music-making across all styles that welcomes contingency in order to enable a distinctive body of work on the composition-improvisation continuum. The assertion of this research is that contemporary trends in ECIC practice are strong and effective, and yield surprising, provocative, and creative results.^9^ As Gottschalk (2016, 1) says when discussing a contemporary definition of experimental music it “is challenging to pin down because it is not a [one] school or a trend or even an aesthetic. It is, instead a position – of openness, of inquiry, of uncertainty, and of discovery.”^10^

### The Relationship Between Improvisation and Composition

In recent years, scholars have investigated more thoroughly the relationship between the processes and outcomes of improvisation, and those of composition. Models researched include those of temporal perception (Sarath, 1996), potentiality (Agamben, as outlined in Lexer, 2010),^11^ equivalence (Rink, 1993; Nettl and Russell, 1998), continuity (Alperson, 1984), complementarity (Siepmann, 2010), interpenetration (Hamilton, 2000), process as product (Sawyer, 2000), and certitude (Peters, 2012). Recent handbooks and readers on the subject include Piekut and Lewis (2016) and Hamilton and Pearson (2020). These musicological, ethnomusicological, psychological, and philosophical studies have provided a greater understanding of the environmental, cognitive, and creative networks involved in improvisation, and the interrelationships between different strata of thought and resulting actions. As a consequence improvisation is now better positioned to be regarded as a complex and malleable process – far beyond a simple “action/reaction” (my words) or cause and response environment – and as an effective expression of composition. Improvisation is slowly and belatedly gaining equal status alongside the score in Western Art music, perhaps, previously delineated due to “the [historical] great divide between low culture and high culture” (Piekut and Lewis, 2016, 5). The contribution of studies by Berliner (1994), Bazzana (1997), Benson (2003), Goehr (2007), and Small (2011), and others, has helped to establish a focus on the importance of the performer and the performance, and redress the cultural value imbalance between the composer and the performer. Benson illuminates the collaboration involved in music making stating “if performers are essentially improvisers, then authorship becomes more complex. That is not to deny composers their respective place as ‘authors’… but it is to acknowledge that their authorship is really a coauthorship, both with those who have gone before and those who come after” (126). Regarding the in-real-time art of improvisation, Hamilton (2007, 213) argues that “improvisation and composition are interpenetrating opposites – that is, features which appear definitive of one are found in the other also.” According to ethnomusicologist Bruno Nettl “musics in the oral tradition do not make the distinction between composition and performance which the concept of improvisation implies” (1998, 11). For Nettl (1974, 6), “the juxtaposing of composition and improvisation as fundamentally different processes is false, […] the two are part of the same idea.” Hamilton (2000, 171) agrees and argues further that such works occupy a continuum and “there is in important respects a fluid contrast between a composed work and an improvisation. Their exemplars stand in a continuum, and ‘improvisation’ and ‘composition’ denote ideal types or interpenetrating opposites.” Both Hamilton and Nettl promote the concept of the composition-improvisation continuum, which allows an infinite range of possible positions between the “ideal types” of the premeditated (and notated) and the immediate (in-the-moment, performed). As an example, composer and improviser Pauline Oliveros refers to improvisation as “speeded-up composition” (Duckworth, 1995, 166). While this may be a prevalent idea among some improvisers, it fails to take into account the unavoidable incidents or accidents, which occur in the moment of performance, and which can and do change the outcome, even if only negligibly.

### The Relationship Between Improvisation and Experimentalism

As mentioned earlier, Lewis has done much to uncover the fundamental interrelationship between improvisation and experimentalism (for example, 1996; 2009). Clearly from the outset, there has been interpenetration of process and outcome in music-making activities described by these categories. In the EM movement, improvisational potential first appeared under the guise of “indeterminism.” Feldman’s score *Projection I* (1950) is an early example of this “open form” type of work. The score is written graphically as a series of boxes and symbols which offer various choices to the performer regarding pitch, playing technique, and duration. This score cannot be interpreted literally and consequently relies on realization by the performer and the opportunity for this to occur in real time (Welsh, 1996). Brown refers to this in-real-time realization potential as “creative ambiguity” (Gresser, 2007, 377). A good example is his early graphic score *December 1952*, a page of horizontal and vertical lines to be performed by any number of instrumentalists, which can be interpreted from any orientation and in any manner that the performer(s) choos(es).^12^ Wolff has had a more openly disclosed relationship between experimentalism and improvisation. He favors “surprise,” “disruption,” and “provocation,” and seeks to ensure that a performer’s improvised contribution to the work does not follow “habitual techniques.” His scores, such as *Duo for Pianists I* (1957), “rely upon the consequences of intentional and non-intended sounds in the performance moment, and the unpredictabilities of ensemble playing” (Thomas and Payne, 2020, 29). Contemporary texts that have investigated the interpenetrations, commonalities, and the continuum between improvisation and experimentalism – both within and also across genre – are by Beins et al. (2011), Piekut (2014), Gottschalk (2016), and DiPiero (2018). Gottschalk, Beins et al., and DiPiero investigate the range, nature, and application of the collaborative process in an indeterminate environment. As Gottschalk (2016, 188) states regarding contingent music-making: “interaction, improvisation, and indeterminancy, these three terms are not interchangeable, but they share a common center: the unknown.” DiPiero (2018, 2–3) explains contingency as “an umbrella term for events that either were or will be decided according to some non-linear causality, a term that is cleaved in half depending on where in a temporal process one chooses to look” and says “contingency invites us to consider every improvisation as non-trivially different – a constellation of openings and closures both, in a singular arrangement.”

In *Tomorrow is the Question* of Piekut (2014) – while not overtly focused on improvisation – explores the development of experimentalism and its various relationships with improvisation in “unexamined” music scenes and cultures, such as music practices in Japan, Cuba, and Bali in the 1960s and 70s, and the stylistic “pluralism” evident in the New York Downtown scene in the 1970s and 80s which saw experimentalism “art music, improvised music, and rock” converge (78). In *Echtzeitmusik Berlin: Selbstbestimmung einer Szene*, Beins et al. (2011) interview composers and improvisers in Berlin and uncover a range of attitudes and approaches toward composition, improvisation, collaboration, and the experimental. Philosophical positions adopted by some result in questions regarding the distinctions between these terms, and the existence or otherwise of contingency and chance within the real-time music making process. These differing points of view highlight the relativity of continua between experimentalism, composition, and improvisation – continua that are recalibrated according to individual aesthetic outlook. It is important to note that improvisation and experimentalism are not necessarily part of the same idea. If a work is more improvisational it may not necessarily be more contingent or experimental. It could be argued that *some* improvisation – such as that which takes place within repertory improvisational styles; perhaps, in some iterations of Indian music or some expressions of jazz music – is so formulated and imitative as to be non-experimental. In these situations, music-making may take place in a highly controlled environment that deliberately limits engagement with contingency, chance, and indeterminism. Similarly some contemporary, adventurous, and non-mainstream work, such as that which Demers describes, may feature neither engagement with improvisation, nor experimentalism. Additionally contingency, indeterminism, chance, or experimentalism might be engaged with by the composer in the process of the composition of a work, but not in its performance. Priest (2013) and Voegelin (2014) expand the notion of improvisation and the associated mental constructs and outcomes in experimentalism to include the audience. Both note that an indeterminate work may offer a range of perceptual possibilities to the perceivers, such that they complete or realize the work through in-the-moment choices made in hearing. Voegelin (2014, 28) draws on the Phenomenological theories of Merleau-Ponty and postulates a “phenomenological possibilism,” that exists in the apprehension of ambiguous sound art works, as “a plurality of actual, possible, and impossible sonic worlds that we can all inhabit in listening” (14). Though not described as such, this in-the-moment aural constitution of the work by the listener is essentially an improvisatory act: a decision or set of decisions made in real time, and contingent on circumstance, that leads either consciously or unconsciously to a perceptual outcome. Consequently, it can be observed that the relationship between experimentalism, composition, and improvisation (ECIC) operates not just in the processes of the physical construction of the musical work but in its apprehension. McAdams (1984, 319) states that “perceiving is an act of composition.” As has been discussed composing in-the-moment or in-real-time is an act of improvisation.

Priest (2013, 22) describes a certain sort of aural “unfocussing” that contemporary urban society is trained in: “the unconsciously acquired habits of listening away and underhearing music,” and notes how this has been manipulated in experimental music, saying “certain contemporary experimental compositions exploit the drifts and digressions of distraction in a way that paradoxically draws attention to the ‘black noise’ and ‘allure’ radiating from musical sounds that have become something to be ‘unfocussed on’” (23). Priest here is describing a form of engagement in which the composer and/or performers and the audience (or perceivers) formulate a musical result based on a contingent listening environment: a result that can be different for each listening subject involved.

### The Relationship Between the Environment and Contingency

The relationship of audience and environment to contingency was sensationally publicized at the premiere of Cage’s *4′:33″* in which the perceptions of the audience and the sounds of the environment were revealed as being under scrutiny, rather than the sounds being made by the performer (1952, see Nyman, 1999, 11). In the 1960s, the Minimalist music extension of EM, as developed by La Monte Young, Reich, Riley, Glass, and others, exploited the environmental context and the perceptions of the audience by directly – or by process – manipulating sounds in the space in which they were activated, thus enabling sonic illusions and psychoacoustic effects. Steve Reich referred to these effects as sonic “by-products” saying

“These mysteries are the impersonal, unintended, psychoacoustic by-products of the intended process. These might include sub-melodies heard within repeated melodic patterns, stereophonic effects due to listener location, slight irregularities in performance, harmonics, difference tones, and so on” (Reich and Hillier, 2004, 35).

The enabling of perceptual possibilities, and the contingencies of environmental interaction, continue to be of interest and offer potential for sonic experimentalists today.

### Experimental Techniques and Processes in Composition, Performance, and Perception

An important aspect of experimental music investigation has been the recognition that contingency and chance act on the music in a variety of ways. There are a number of techniques, mechanisms, processes, and actions which take place, or are activated by composers, performers, listeners, and environments to bring about the experience of contingency in music. Contingency and chance can act on, or be acted on, by the composer or performer prior to the music-making event. This might be due to: the adoption of chance or process-based compositional procedures, deliberate “forgetfulness” in compositional and/or performance practice process (what Priest, 2013, 18 calls “intentional unintentionality,”; see also Feldman’s composition process in Feldman and Friedman, 2000), or the collection or rehearsal of contingent music-making procedures (Bailey, 1993).

Additionally, contingency and chance can act on, or be acted on, in the music-making by a single performer or part or whole of the ensemble. This might be due to: deliberate or unintentional forgetfulness, long-form performance of persistent repetitions of limited musical material (that may be sounding in and out-of-phase with other performers’ sound-making), deliberate destabilization of sonic continuities (Thomas and Payne, 2020), instrumental preparations or electronic sound-making processes, exploiting environmental resonances and psychoacoustic effects, and seeking to hide or reveal form and content by dynamic means (Hegarty, 2007; Beins et al., 2011). These elements or forces can also act on, or be acted on, by the audience or perceiver of the music-making – this might be due to: distraction or daydreaming, musical taste, deliberate listening choices, imagination, and physical impairment (Voegelin, 2014) – and by the environment of the music-making. This might be due to: the shape of the performance space, the presence or lack of sound reinforcement and amplification, the position of the listener in the space, the dynamics of the musical performance, the sonic content of the musical performance, and the placement of the performers (Conrad, 1997).

## Methodology

### The Experimental Composition Improvisation Continua

I contend that the historical definition of experimental music as “an act the outcome of which is unknown” remains a useful descriptor of music that deliberately engages with contingency and chance events, such as that which can exist in expressions of jazz, or expressions of field recordings, or prepared instrument practice, or any of today’s innumerable contemporary stylistic outputs. Nyman outlines a continuum in experimentalism as follows:

“The extent to which they [musical processes/acts/outcomes] are unknown (and to whom) is variable and depends on the specific process in question. Processes may range from a minimum of organization to a minimum of arbitrariness, proposing different relationships between chance and choice, presenting different kinds of options and obligations” (1999, 4).

**Figure 1:**
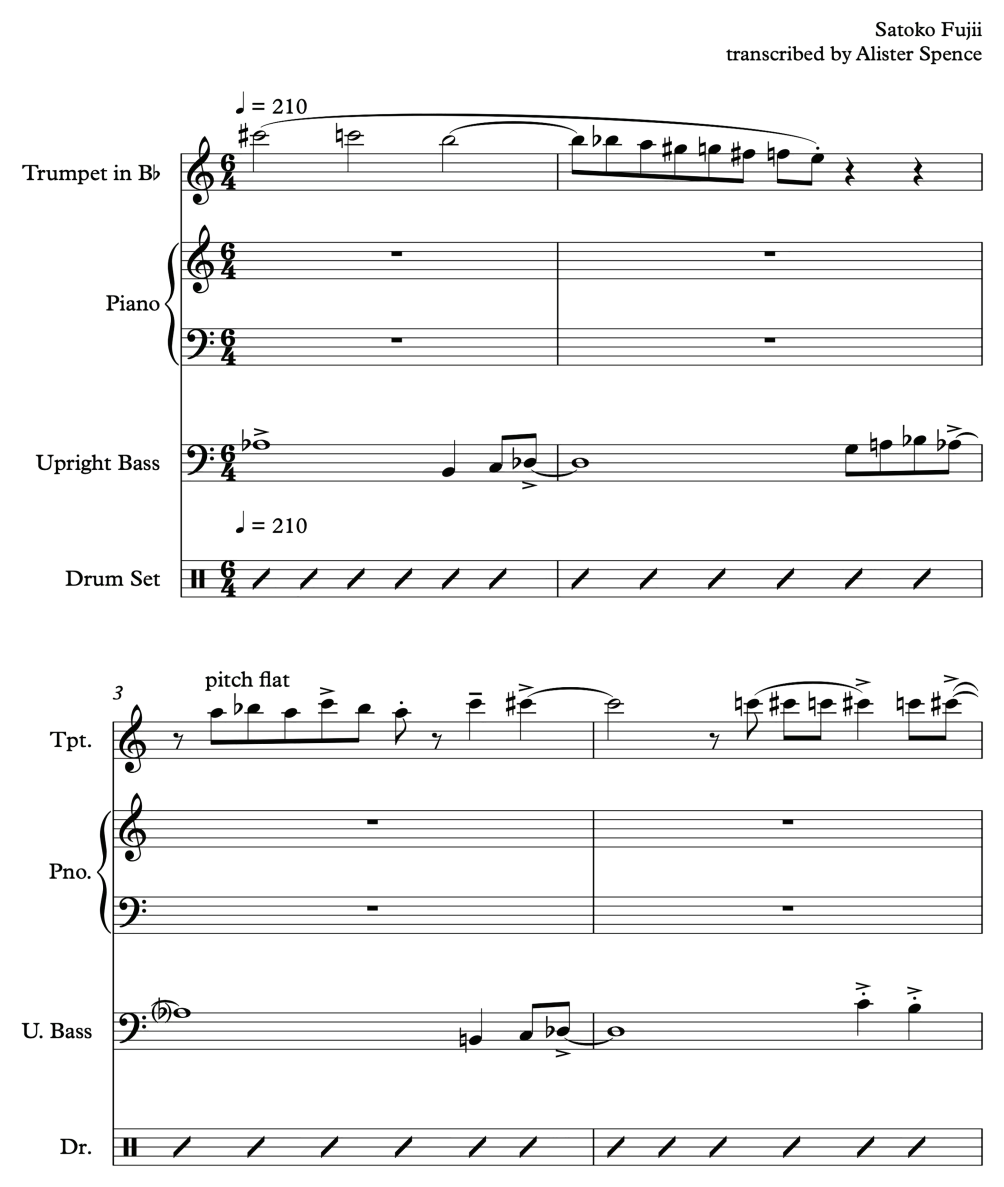
The Experimental Composition Improvisation Continua (ECIC).

I offer the *ECIC* as a model by which to investigate interactions in musical works between relative experimentalism on the one hand, and composition and improvisation on the other.^13^ The diagram in Figure 1 shows what is essentially a field having two axes, the experimental axis and the composition-improvisation axis.

The composition-improvisation axis represents an infinite range of possibilities between the “ideal types” of the completely composed (i.e., premeditated, notated, or scored) and the completely improvised (i.e., in-the-moment, in-real-time, and performed). The experimental axis represents an infinite range of possibilities of musical activities from the “ideal types” of not experimental (i.e., having no engagement with contingency/chance/indeterminism) to completely experimental (i.e., completely engaged with contingency/chance/indeterminism).

The ECIC model makes it possible to consider, observe, track, or plot, musical activities and relationships. Any musical work can occupy any position on the continua field, at any time; and choices that are made or allowed, either physically or psychologically, will steer the work toward a particular mix of the three elements – composition, improvisation, more or less experimental – and a particular position on the ECIC field.

Composers and improvisers can and do adopt *various positions* along these continua, including at various times in their careers, and for a variety of reasons (for example, see Wolff, in Lucier, 2018, 12-30). Also within a single work, as mentioned, positioning may vary as it progresses. Similarly, the receivers of the musical work – the audience (or listeners, or perceivers) – may choose or occupy various perceptual positions within the span of one work, or adopt various listening positions across the years of their many and various musical engagements (Cook, 1990).

As a tool, the ECIC model must be employed relatively according to the user and context. Each person who employs it will bring their own set of assumptions and predispositions to the musical situation(s) being analyzed. The ECIC model provides a means to consider the various *qualitatively* apprehended engagements within and between experimentalism, composition, and improvisation, in musical works.

### ECIC in Practice: An Analysis of Three Works

In the next section, three distinctive works are investigated using the ECIC as a tool in order to illuminate the presence or otherwise of various experimental interactions within them. The first work is “Spiral Staircase (SS),” a composition by written by Satoko Fujii in late 2007.^14^ The second work is John Cage’s *4′33″*, and the third is a 1965 performance of “My Favorite Things” by the John Coltrane Quartet.

## Results

### “Spiral Staircase”

**Figure 2:**
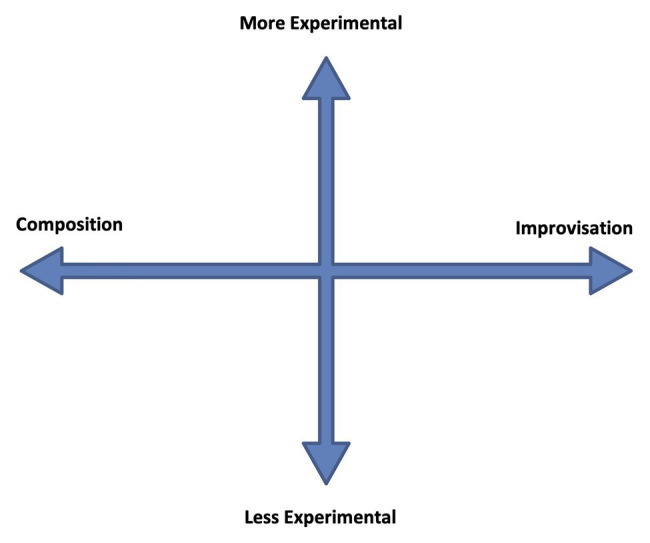
“Spiral Staircase” melody excerpt (full score).

The recorded work referred to here is from an audio CD called *Heat Wave*, performed by “Ma-Do” quartet of Fujii (2008). The performers are Satoko Fujii (piano), Natsuki Tamura (trumpet), Norikatsu Koreyasu (double bass), and Akira Horikoshi (drum kit). I am using the term work, in this case, to refer to one specific performed and recorded iteration: an “event.”^15^ It is common practice in jazz and improvised music that, though one person may be afforded the composer credit for the work, it is understood the particular iteration is indebted to all performers as they are also compositional contributors, informing the final product. “SS,” the work, is 4 min 25 s long and consists of a combination of composed and improvised sections. These sections are able to be detected aurally, *via* repetitions of complex material, pitch, and rhythm associations, and other changes in the sound as the performers interact. The composed material can be detected due to the presence of near-exact repetitions of passages of complex melodic and rhythmic material restated at various points throughout the work. ECIC interactions are evident from the beginning moments of this work. The first 34 s are occupied by the initial statement of the melody, with each performer playing a specific, pre-composed part on their instruments. However, from a perceptual point of view the nature and combination of the composed musical parts is stylistically, temporally, and formally ambiguous. The melody statement, played in unison on the trumpet and piano, sounds as a convoluted stream of eighth notes with an indecipherable metric structure that is interrupted at irregular intervals by low pitched sounds played with a loud dynamic in rhythmic unison on the piano, double bass, and drums, as shown in Figure 2.

**Figure 3:**
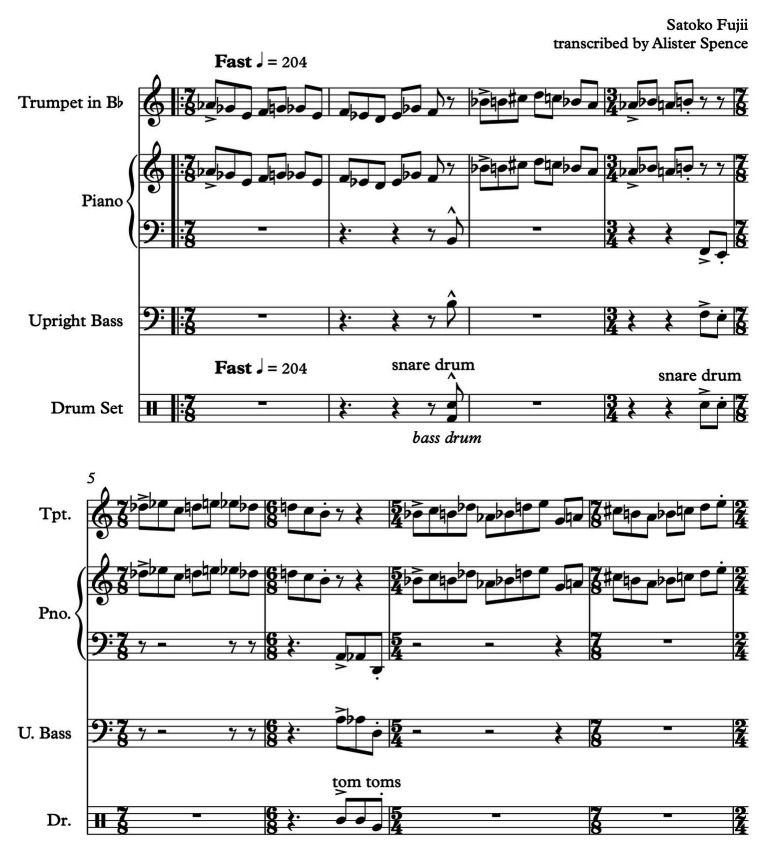
ECIC interactions in “Spiral Staircase.” The asterisk indicates the position of events: A, and/or B, and/or C, and /or B1, etc.

This construction stimulate an indeterminate, contingent, listening environment, as the progress and outcome of the music neither cannot easily be traced or predicted, nor can a patterned relationship be determined between the music’s parts. Stylistically, it sounds as if derived not only from jazz, but also, contemporary classical music, rock, and Okinawan court music (Spence, 2018). The ambiguity of form, content and, to some degree, style, encourages the listener to disengage expectation. If this section of the work were to be located on the ECIC field, a possible location would be toward the composition periphery of the composition/improvisation axis, due the sounding chiefly of pre-composed elements; and on the more experimental side of the more/less experimental axis, due to the perceptual indeterminism that the music engenders. In Figure 3, I have indicated this as a relative positioning marked with the letter “A” as it is the first ECIC event consideration in the timeline of the work.

### The Piano Improvisation

**Figure 4:**
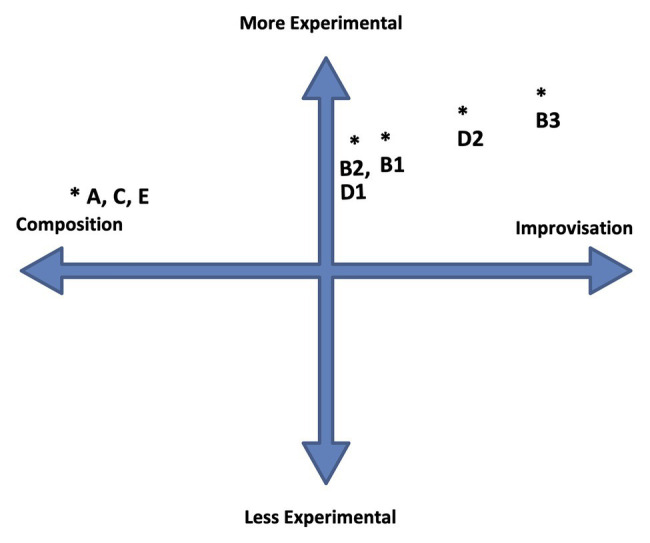
“Spiral Staircase” piano improvisation and accompaniment excerpt (full score).

The next section of the music that follows – here referred to as section B – contains the sounds of Fujii’s improvisation on the piano and some sporadic accompaniment played at the beginning by Koreyasu and Horikoshi on the double bass and drum kit. It is 1 min and 40 s in duration and quite complex with respect to ECIC relationships. For a short period, the bass player and drummer play apparently pre-composed/notated rhythm and pitch interjections (notated by Fujii), in close-to-but-not-quite rhythmic unison. For the listener, it is very difficult to apprehend the logic behind the patterning of these interjections due to the temporal space between events and lack of audible metric pulse. Additionally, Fujii is playing quite different rhythms to those of Koreyasu and Horikoshi in her piano improvisation, and with an alternate, also ambiguous, metric association. These are shown in Figure 4.

The perceptual indeterminism of the accompaniment and its relationship to the pianist’s improvisation is heightened by the seemingly indeterminate improvisational approach adopted by Fujii, who constantly changes rhythmic patterns and melodic material as her improvisation continues. The apparent arbitrariness of rhythm-pitch-melody relationships, the out-of-time asynchronous nature of the piano performance compared to that of the bass player and drummer, and the approximately timed, irregular iterations of the (composed) bass, and drum accompaniment, introduce elements of physical, performance indeterminism to the ECIC environment. For the performers, there is a sense of the unknown related to their performance; as to how, and when, and in what manner, it will interact with that of the other band members. Performance indeterminism combines to augment the already existing perceptually contingent listening environment. Point B2 (as indicated on Figure 3) shows a possible relative placement of this section of “SS” on the ECIC field: the indeterminate nature of the performance of composed elements combined with the improvisational focus of the music locates this point to the right of the center of the composition/improvisation axis. And the augmented contingent environment, due to physical as well as perceptual factors, accounts for this point being located further toward the more experimental periphery of the more/less experimental continuum than point “A.” There are further musical developments in the piano improvisation section (B) that contribute to the perception of a change of position within the ECIC field. After 24 s from the beginning of this section, the bass player and drummer adopt an obvious, easily recognizable metric rhythmic accompaniment which lasts for 16 s. This doesnot seem to sway Fujii from her physically contingent course, however, it does steer the work perceptually more toward an equal distribution of composed and improvised elements. Point B2 (Figure 3) shows a possible location of this musical passage on the ECIC field. Following this, the bass player and drummer stop playing and Fujii’s improvisation becomes more varied, as she incorporates a wider instrumental range; playing faster chromatic passages, and dividing the performance of the constantly changing rhythmic patterns between both hands, once again in a seemingly random fashion. This is an expressionistic, gestural approach such as that adopted by American free jazz pianist Taylor (1973), for example, on his piece “Indent: Second Layer” (analyzed in Westendorf, 1994, 125–155). These are gestures in which the shape of the improvisation is marked out in a general way by physical, “in-the-moment,” un-premeditated actions. The resulting sound/pitch content of the improvisation is arbitrary and contingent on these actions. This third development within the B section of “SS” is marked as B3 on the ECIC field diagram. The perception of this last section of Fujii’s improvisation is that the level of engagement with contingency and chance is increased due to bolder, arbitrary physical action, with no constraining or comparative structure (such as that provided earlier by the accompaniment of the bass player and drummer), and with highly contingent sonic results. At this point, the work is also perceived as having moved closer toward the improvisation periphery of the continuum. Following the piano improvisation, there is a re-statement of the melody played in a nearly identical fashion to the opening statement of the work. This is indicated on Figure 3 as section C and is located on the ECIC field in the same place as point A for the same reasons as those listed earlier.

### The Trumpet Improvisation

**Figure 5:**
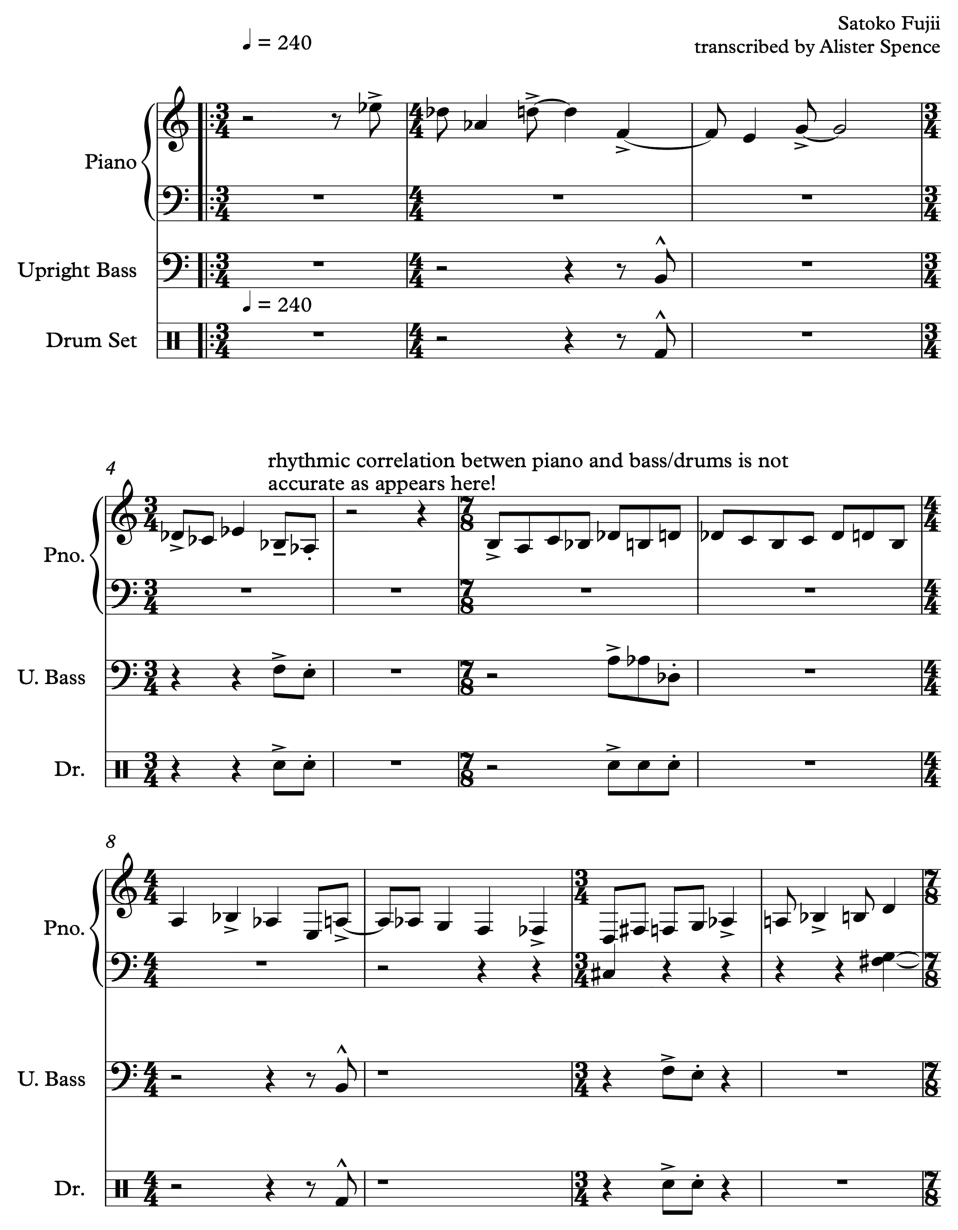
“Spiral Staircase” trumpet improvisation and accompaniment excerpt (full score).

Tamura’s trumpet improvisation follows (see Figure 5). This section of the music – called Section D in this analysis – is 52 s in duration. Like Fujii, Tamura appears to form arbitrary, chromatic, and pitch relationships in his improvisation, though initially within a more restricted range. This time, the bass player and the drummer play a repeated rhythmic and melodic motif, with an easily discernible metric underpinning, that continues for the duration of the trumpet improvisation. This is indicated as point D1 on the ECIC field diagram at the same location as B2, and for similar reasons, due to the more or less equal distribution of composed and improvised musical elements combined with the physically and perceptually contingent nature of the trumpeter’s improvisation. Once again, as with the pianist’s improvisation, there is an apparent shift of position on the ECIC field as Tamura’s improvisation progresses. This time, it is due to the entrance of Fujii, 20 s after the start of the trumpet improvisation, who plays dissonant, constantly changing chord patternings on the piano that are out of time with the bass and drums and unrelated harmonically to the sounds being made on the trumpet. This extra indeterminate element in the music shifts its position on the ECIC field as perceived by this listener. In Figure 3, it is shown as point D2. The work finishes as it started with a further statement of the composed “SS” theme. This section is shown on the ECIC field as point E, located once again in the same place as point A.

### Summary of Factors in “Spiral Staircase” Affecting ECIC Positioning

Experimental Composition Improvisation Continua interactions are discernible in “Spiral Staircase” due to a range of factors. These factors include:

- Austerity and ambiguity of compositional style which require performers to interact in real-time to develop and extend the material.
- Irregular bass register interjections (in the melody sections of “SS,” and in the piano improvisation), which deliberately disrupt narrative flow and destabilize apprehension of tonality, meter, and form.
- A performance approach that welcomes approximation or inaccuracy: as heard when the bass player and drummer are initially playing the accompaniment to the piano improvisation, or when Fujii deliberately ignores meter while accompanying Tamura’s improvisation on the piano.
- Improvisational style which favors ambiguous rhythmic gesture, sudden change, juxtaposition, and welcomes indeterminism of pitch and time.
- Perceptual indeterminism due to ambiguous musical form and content, and the nature of the improvisation and contingent musical interplay between performers.

### Performer Action vs. Listener Experience in *4΄33″*

**Figure 6:**
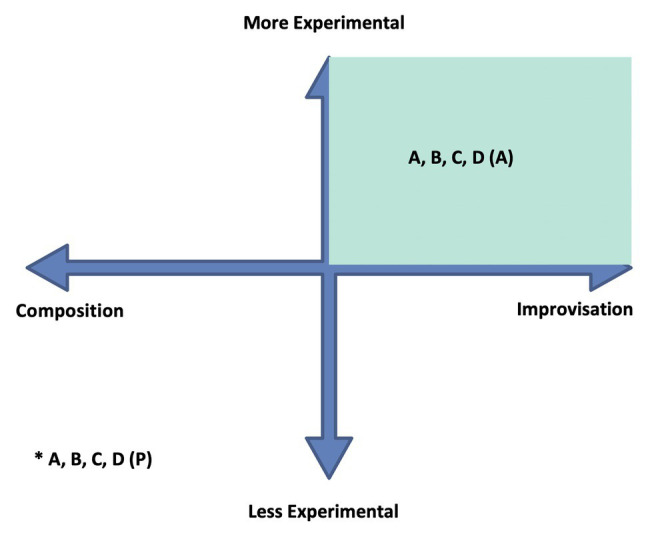
*4′33″*: ECIC diagram showing performer and audience relationships. The asterisk indicates the performer’s position on the ECIC at points (sections) A, B, C, and D, as referred to in the text.

To demonstrate how the ECIC can be applied further, and to existing well-known works let’s examine Cage’s (possibly most famous) composition *4′33″* as performed by a solo pianist. This work is interesting from the point of view of investigating engagement with contingency and chance in a work, and the contrast between the experiences of the performer and the audience in its realization. *4′33″* relies on the audience and performer being together in the performance space. The piece is divided into three sections of varying lengths: 30 s; 2 min, 23 s; and 1 min 40 s, respectively. The actions generally employed by the pianist in this piece have evolved from those adopted in David Tudor’s premier performance in 1952. They are as follows: to open the lid at the beginning of each section and close it at the end, before opening again for the next section (Holzaepfel, 2001, 2).^16^ The performer needs to pay careful attention to a timekeeping device to ensure the sections of the piece are correct length. Figure 6 shows with an asterisk a possible location of points A–D: where A is the point in the piece where the lid is lifted for the start of the first section, B is where the lid is closed then opened again for the second section, C is where the lid is closed then opened again for the third section, and D is where the lid is closed for the end of the third section and conclusion of the piece. The “P” in brackets indicates “performer.” Because these actions are almost identical in terms of their relationship to composition, improvisation, and experimentalism, they are located with one asterisk toward the more composed and less experimental extremities of the ECIC model diagram. The nature of the audience’s listening engagement in a performance of *4′33″* is central to the work. While the performer’s role is essentially to enact concrete physical tasks (albeit without playing the piano keyboard), the audience’s experience is dependent on their imagination. As this work is now well-known, most audience members will have some idea what to expect, however, when the lid of the piano is opened for the first time and no sound on the instrument is made they need to determine what constitutes this piece for them. They can engage in this listening/realization process systematically, or creatively, or distractedly, or dismissively; but regardless of their approach they will be operating in the more experimental (contingent, chance dependent, and indeterminate), improvised (in-the-moment, in-real-time) quadrant of the ECIC model, as no sonic result that constitutes *4′33″* exists before they “compose” it in the moment (McAdams on perception as mentioned earlier). This is shown as a shaded area on the model in Figure 6 to indicate the location of the myriad of possible perceptual choices or experiences in the realization of the work. The bracketed “A” letter indicates “audience.”

### The Sound of Effort and Experimentalism in the John Coltrane Quartet, 1965

**Figure 7:**
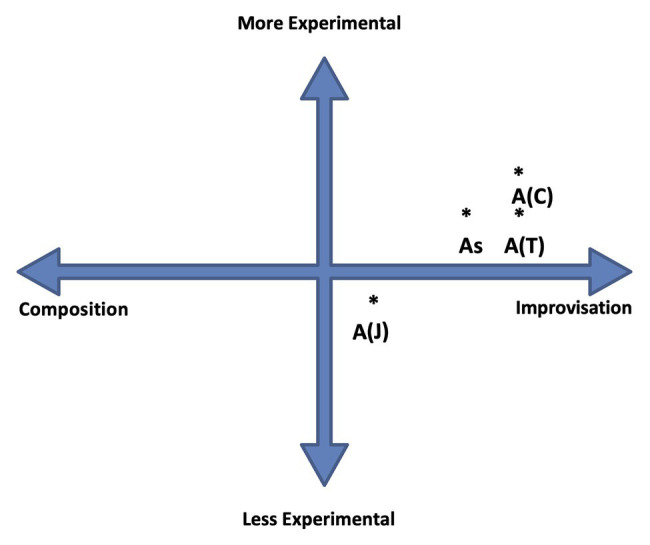
ECIC relationships in Coltrane’s solo on “My Favorite Things.” The asterisk indicates the position on the ECIC of the individual performers in Section A of the performance of “My Favorite Things,” as referred to in the text.

On August 1, 1965, John Coltrane played a concert at a jazz festival in Comblain-la-Tour, Belgium with what was called his “classic” quartet: Coltrane (tenor and soprano saxophone), McCoy Tyner (piano), Jimmy Garrison (double bass), and Elvin Jones (drums). By this time, the band had been playing together constantly for 3 years, and Coltrane was becoming more interested in free jazz. In early 1966, Coltrane recorded *Ascension*, a distinct move in this direction (see Jost, 1994). However, in 1965, the quartet’s repertoire still included “show tunes” – popular tunes adapted to jazz. The quartet’s performance of “My Favorite Things” at this concert offers a means to study ECIC relationships in this ensemble, particularly in connection with effort and gesture. Coltrane first recorded “My Favorite Things” on an album of the same name in 1961, and the quartet had played it regularly since then (Coltrane, 1961). The band’s performance dynamic by this time was extremely energetic. As Ashley Kahn describes in the liner notes to the recording: “it was what the quartet delivered in extended doses: sustained, elevating energy and a marked density of musical ideas, a heightened sense of drama, and a sweat-inducing delivery that seemed to somehow articulate answers to spiritual mysteries. It was hip and hypnotic, frenetic, and at times frightening’ (2007). As this quote indicates there was a sense in the ensemble, and among contemporary jazz music performers of the time, that playing at the limit of one’s abilities was a means to discovery and the players worked hard musically to encourage each other in this quest. In musical terms, these passages of extreme effort cause or allow the most indeterminacy in this performance; either due to the individuals being close to their technical limits, taking risks and uncertain of the musical outcomes, or because of the combined sonic effect of the quartet’s performance. This demonstration is the beginning of an investigation of ECIC relationships in Coltrane’s soprano saxophone solo which is heard almost 131/2 min after the beginning of the work and continues for almost 3 1/2 min (Figure 7). The work is based on motoric repetitive rhythm (over a 3/4 m) and at this point a repeated harmonic motif – one bar each of Emi7 chord then F#mi7 – underpins the whole saxophone solo. Tyner, Garrison, and Jones are performing as accompanists and have several roles: to maintain these underlying rhythmic and harmonic structures, to embellish and develop musical ideas from it, and to interact with the soloist and encourage them in their musical explorations. At time, the pianist and drummer are momentarily defaulting to pre-determined (composed) rhythmic and harmonic patterns that were played in the melody section of the work (and on performances previously). In this recording, the major changes in dynamics, interactivity, and musical texture are driven by Coltrane, Tyner, and Jones. Garrison’s contribution, while not insignificant, has been excluded from this diagram. Four periods of group effort and gesture intensity have been identified in the almost 3 1/2 min saxophone solo. These are the points at which the music’s relationship to experimentalism is most active. Each player has a slightly different or changing relationship with experimentalism (contingency/chance/indeterminism), composition, and improvisation, in these sections. For the purposes of this demonstration, I am only investigating what I have called section A, which begins 7 s after the beginning of Coltrane’s saxophone solo. Section A occurs from 13′27″–13′39″ in the work. At this point Coltrane, who was playing short 16th note passages plays two 4-bar extended continuous chromatic 16th note passages, and Tyner modulates his chord voicing structures on the piano freely, in a seemingly random fashion, and in a vigorous contrapuntal manner, over a wider range of the instrument. Jones maintains the ongoing motoric rhythmic feel on the drums punctuated by one beat “fills” (embellishments) at the end of each bar. There is certainly musical tension here but there is also risk, uncertainty, contingency, heard in the occasional spectral splitting of the saxophone pitches, and the occasional indistinct chord played on the piano as the players struggle to maintain a coherent musical pathway. On the ECIC diagram, each player relationship with experimentalism, composition, and improvisation in this section is listed by the letter A followed by the initial of performer’s surname in brackets. The overall perceived composite sonic result of the section is indicated by a lower case “s.” The position of A on the diagram for Jones is more toward the composition end of the continuum but still within the improvisation half of the diagram, and less experimental due to less engagement with contingency and chance. Tyner and Coltrane are perceived as equally engaging with the more improvised end of the continuum, and Coltrane slightly more engaged with experimentalism due to the rapidity and chromaticism of the passages he is playing over the full register of the soprano saxophone. The overall sonic result is considered in this case as an approximation based on the various performances. The relationship with experimentalism is also affected by the short duration and regularity of this eight-bar exploratory section. As the solo progresses these sections become longer and the players more involved in interaction. Tyner and Jones take more risks in their performance and the sound of the ensemble begins to become one indeterminate entity, albeit within the restricted parameters of a motoric jazz performance in E minor. Section B is from 13′44″–14′08″; Section C from 14′09″–15′07″; and Section D 15′24″–16′08″. If musical relationships for these sections were to be plotted on the ECIC diagram, we could observe the changes in engagement with experimentalism, composition, and improvisation (This might be best illustrated with four separate ECIC diagrams to avoid overcrowding of information). Following this, the quartet settles into more regular rhythmic, harmonic, and melodic patterns, and Coltrane prepares to repeat the melody to “My Favorite Things.”

## Conclusion

There are a range of musical situations where the ECIC model might be applied. These situations involving composer, performer, perceiver, and environment can occur within the formation and realization of the work. As outlined ECIC considerations can be useful when determining the sources of contingency and indeterminism: whether this is due to the environment in which the work is performed, or due to the compositional or improvisational style, or performer action and interaction. However, ECIC considerations can also be of assistance when comparing musical works. These works can be similar or different in style and content (Spence, 2018). Applying the model helps to answer questions such as is contingency and chance the main focus of a musical work, or is it a by-product of the events that enable it (Spence, 2018)? Is indeterminism caused by physical action (human or otherwise) due to laws of physics, or is it psychologically activated; or a combination of both (psychoacoustic)? The ECIC model can also be of assistance when comparing a composer’s stated aims or compositional approach or process, with the actual or perceived sounds of their work. For instance, indeterminism might be detectable, using the ECIC model, as being more or less at play in the composer’s work than they are aware of, or that they indicate in published statements (Spence, 2018). Similarly, the composer’s compositional technique can be examined through the lens of the ECIC. Do they employ chance techniques in order to complete a score (this can involve improvisational techniques as well as the use of externally imposed processes such as those made famous by Cage)? And to what extent is this perceivable in the sound of the compositional result? Do they consider the score as an end point, a blueprint for correct performance, or simply as a catalyst, or suggestion: a means to engage performers in the realization of a musical work? A performer’s intentions and actions also can be investigated with reference to the ECIC model. For instance to what degree does their improvisational output demonstrate reliance on pre-learned (“pre-composed”) motivic patterns, or in what ways do they deliberately, or by process, de-stabilize their performance environment in order to engage with contingent events as a driver for discovery and new ideas? How is their output affected by interactions with other performers, or the score, or the environment? This article has investigated the historical and ongoing relationship between experimental music, composition, and improvisation, and shown that while there are clearly identifiable interpenetrations between the practices, processes, and outcomes expressed in these musical approaches, there are nevertheless distinctions that characterize their ideal types. By identifying these distinctions and the continua between them, a model for experimental music investigation has been developed: the *ECIC* model. This model offers a way investigate and compare the action and operation of contingency, chance, and indeterminism, on composition and improvisation and the continuum between them. The application of the ECIC model to “Spiral Staircase,” *4′33″*, and “My Favorite Things,” demonstrates how one can more clearly appreciate the relationships between experimentalism, composition, and improvisation in these works, and from a variety of viewpoints, regardless of musical style. Considering a work from an ECIC perspective can help to identify points at which experimentalism, composition, and improvisation are more or less activated, and in comparison to other sections in the work; or compared to a composer’s previous output; or compared to the “norms” of a musical style, or cultural approach. As indicated in the discussion of “My Favorite Things,” multiple iterations of ECIC model diagrams can be applied to the one work. With longer works, different iterations can be dedicated to the various sections of the work. This can help to avoid overcrowding the ECIC field and for easier comparison between sections. Contingency and indeterminism are at play in almost all music, even when least thought to be active, such as in the performance of a “completely composed” music score. Here, also a continuum continues to be present between composition and improvisation (Bazzana, 1997). A case could be made for applying the ECIC model to all musical actions and outcomes. However, the ECIC has most relevance as an investigative and comparative tool in experimental music, where contingency is deliberately welcomed as a catalyst for new musical ideas and unknown outcomes. In this environment, as has been previously stated, the relationships between contingency/indeterminism/chance, composition, and improvisation are constantly in flux. In experimental, music, the ECIC model can be used to observe and investigate music composition, performance, and perception; across style, and scene, and culture; and the drivers for music, “the outcome of which is unknown,” can be traced, isolated, and compared.

## Data Availability Statement

The original contributions presented in the study are included in the article/supplementary material; further inquiries can be directed to the corresponding author.

## Author Contributions

The author confirms being the sole contributor of this work and has approved it for publication.

### Conflict of Interest

The author declares that the research was conducted in the absence of any commercial or financial relationships that could be construed as a potential conflict of interest.

## Footnotes

^1^It should be noted that in this article, I am referring the term “experimental music,” and what centrally defines it, as outlined by its historically situated practitioners in the Experimental Music movement in the 1950s and 60s.

^2^Two examples of a broad overview of contemporary experimental work are *Experimental Music Since 1970* (Gottschalk, 2016), and *Audio Culture* (Cox and Warner, 2013). Piekut also investigates cross-genre; cross-culture experimentalism in *Tomorrow is the Question* (2014).

^3^In this article, I take “work” to also include the sounds that result due to performance, which may be linked to pre-existing compositional material (“scores”). As Max Neuhaus states “These pieces [scores] are not musical products, they are meant to be activities” (Goehr, 2007, 244). Goehr points out that the concept of the musical work is an open one and has been adopted and adapted in ways that “extend the concept’s employment” to refer to musical styles and cultural settings well beyond its classical music origins and including improvisation (255). Benson states “what we call a ‘work’ might better be thought of as a developing structure that arises from the activity of music making” (2003, 147).

^4^Since Lewis, McMullen has suggested “Asialogical” as a further socio-cultural musical delineation in improvised music (2003, cited in Borgo, 2002, 171).

^5^For background on Brown and jazz, see Yaffé (2007, 289-290). For Riley see Duckworth (1995, 266), and La Monte Young see Duckworth (1995, 210).

^6^*Indeterminism* is defined by the *Oxford Dictionary* as “the doctrine that not all events are determined by antecedent causes” (Stevenson, 2010). In a musical setting, indeterminism can be engaged with by composers, performers, and the audience with the resultant outcome determined by the in-the-moment decisions made by the participants in direct relationship to the contingencies of their environment. *Performer indeterminism* entails offering the performer(s) individual choices, usually within certain constraints. For instance, at a certain point in time, the performer chooses freely from a (possibly pre-determined) range of pitch or timbre options (indeterminism of pitch and timbre), or alternatively plays a set order of pitches (or timbres) at a time of their choosing and with any tempo within a (possibly pre-determined) span of time (indeterminism of time).

^7^The term “acousmatic” was employed by Musique Concrète pioneer, Pierre Schaeffer, to support his concept of ‘sound objects’ – sounds perceived independently of their “methods of production and transmission,” sounds heard as unique entities (Schaeffer, 2017, 64).

^8^An example of one such debate between the London free improvisation scene, and the “Reductionists” (or New London Silence) regarding gesture in experimental music can be found in Bell (2005, 32-39).

^9^I use the term “practice” here to refer to a musician’s mode of operation, for example, a “field-recording” artist or a “minimalist jazz” composer and improviser.

^10^For the purposes of this research, I am referring to the following definitions taken from the Oxford English Dictionary: “Improvisation, n.: The action or fact of composing or performing music, poetry, drama, etc., spontaneously, or without preparation; this method of performance” (Oxford English Dictionary Online 2020). “Contingency, n.: The befalling or occurrence of anything without preordination, chance, fortuitousness. …The quality or condition of being subject to chance and change, or of being at the mercy of accidents” (2020).

^11^Lexer says of potentiality in improvisation “This potential is embedded and constituted within the relationship between the performer and the instrument. Thus, it can be argued that the performer and instrument form an intrinsic unit within which previously acquired skills, positive and negative experiences, and intuitively felt possibilities and limitations are manifested through the performer’s personality, motivation, and creativity. This highly complex constellation of human imagination and instrumental possibilities is conceptualized within a potentiality space: A space populated with potential approaches, processes, and responses” (2010, 42).

^12^Brown had a vexed relationship with indeterminism. Over the course of his composition career, he moved away from unrestricted open form works in order to try to preserve what he understood as their identity (Gresser, 2007). Cage similarly sought to restrict interpretive scope and maintain artistic control by working closely with the same performers such as the pianist David Tudor, who essentially became a co-composer with Cage (Holzaepfel, 2001).

^13^As previously discussed there have been studies which investigate a continuum between composition and improvisation, and those that investigate degrees of engagement with experimentalism; however, none so far that bring these continua together in a model.

^14^Fujii is an internationally recognized Japanese improvising pianist and composer, and a colleague with whom I have collaborated and performed since 2007. Regarding peer review see, for instance, Fujii’s recorded work listed twice in the American jazz journal Downbeat (2020): The Year’s Top-Rated Albums’ (2020).

^15^Benson, while not disagreeing with the use of the word “work” for improvised realisations, also suggests the word “piece” as an alternative (2003, 132).

^16^On the score, Cage has indicated that this piece can be played by any number of instrumentalists and for any length of time; however, the practice most adopted for solo piano follows that of the premier performance by David Tudor. It was Tudor, not Cage who “composed” the opening and closing of the piano lid for his performance of the premier of *4′33″* at the Maverick Concert Hall on August 29, 1952. This practice has remained as an integral part of the composition (Holzaepfel, 2001, 2).
